# Target Role of Monocytes as Key Cells of Innate Immunity in Rheumatoid Arthritis

**DOI:** 10.3390/diseases12050081

**Published:** 2024-04-25

**Authors:** Diana I. Salnikova, Nikita G. Nikiforov, Anton Y. Postnov, Alexander N. Orekhov

**Affiliations:** 1 Laboratory of Oncoproteomics, Department of Experimental Tumor Biology, Institute of Carcinogenesis, Blokhin N.N. National Medical Research Center of Oncology, 24 Kashirskoe Highway, 115522 Moscow, Russia; 2 Laboratory of Angiopathology, The Institute of General Pathology and Pathophysiology, 8 Baltiyskaya Street, 125315 Moscow, Russia; nikiforov.mipt@googlemail.com (N.G.N.); ano.inat@mail.ru (A.N.O.); 3 Laboratory of Cellular and Molecular Pathology of Cardiovascular System, Federal State Budgetary Scientific Institution “Petrovsky National Research Centre of Surgery”, 3 Tsyurupa Street, 117418 Moscow, Russia; anton-5@mail.ru; 4 Center for Precision Genome Editing and Genetic Technologies for Biomedicine, Institute of Gene Biology, Russian Academy of Sciences, 34/5 Vavilova Street, 119334 Moscow, Russia; 5 Institute for Atherosclerosis Research, Osennyaya Street 4-1-207, 121609 Moscow, Russia

**Keywords:** monocyte subsets, macrophages, innate immunity, rheumatoid arthritis, chemokines, proinflammatory cytokines

## Abstract

Rheumatoid arthritis (RA) is a chronic, systemic, and inflammatory autoimmune condition characterized by synovitis, pannus formation (with adjacent bone erosion), and joint destruction. In the perpetuation of RA, fibroblast-like synoviocytes (FLSs), macrophages, B cells, and CD4^+^ T-cells—specifically Th1 and Th17 cells—play crucial roles. Additionally, dendritic cells, neutrophils, mast cells, and monocytes contribute to the disease progression. Monocytes, circulating cells primarily derived from the bone marrow, participate in RA pathogenesis. Notably, CCR2 interacts with CCL2, and CX3CR1 (expressed by monocytes) cooperates with CX3CL1 (produced by FLSs), facilitating the migration involved in RA. Canonical “classical” monocytes predominantly acquire the phenotype of an “intermediate” subset, which differentially expresses proinflammatory cytokines (IL-1β, IL-6, and TNF) and surface markers (CD14, CD16, HLA-DR, TLRs, and β1- and β2-integrins). However, classical monocytes have greater potential to differentiate into osteoclasts, which contribute to bone resorption in the inflammatory milieu; in RA, Th17 cells stimulate FLSs to produce RANKL, triggering osteoclastogenesis. This review aims to explore the monocyte heterogeneity, plasticity, antigenic expression, and their differentiation into macrophages and osteoclasts. Additionally, we investigate the monocyte migration into the synovium and the role of their cytokines in RA.

## 1. Introduction

RA is a chronic, systemic, and autoimmune disease. The mechanisms of this condition are still being investigated. The illness is characterized by an inflammatory process in the synovial joints, which is called synovitis [1]. Moreover, RA is accompanied by the hyperplasia of the synovial tissue. The disease is complicated by the destruction of the cartilage structure and the formation of pannus [2]. Throughout the course of the pathology, bone erosion occurs and predominantly surrounds the peripheral synovial joints. The clinical symptoms of RA include pain, edema development, and tenderness of the synovial joints located symmetrically and peripherally [3]. A great deal of progress has been made in the treatment of RA with antagonists of proinflammatory cytokines, such as tumor necrosis factor (TNF), interleukin-6 (IL-6), and IL-1β. However, the disease remains refractory in some patients. Studies have demonstrated that prostaglandins, leukotrienes, reactive oxygen species, nitric oxide, lipoxins, and platelet-activating factors as small-molecule inflammatory mediators play an important role in RA development. Such compounds assist in inducing, maintaining, or reducing inflammation and could therefore be potential therapeutic targets [1].

Innate and adaptive autoimmune cells promote the continuation of RA pathogenesis. Among them are B cells and CD4^+^ T cells, which include the T helper 1 (Th1), Th2, and Th17 cells. Moreover, monocytes/macrophages and resident macrophages participate in the disease development [4]. In addition, synovial cells and FLSs contribute to RA. Furthermore, dendritic cells (DCs), neutrophils, mast cells, and immunoregulatory cells, for example, regulatory T (Treg) cells, are involved in the complex interaction that leads to the preservation of chronic inflammation [5]. The above-mentioned cells stimulate the formation of the modulators of local inflammation, such as cytokines, chemokine ligands, and the appropriate receptors. These modulators take part in synovial inflammation and destruction [6].

Monocytes are a type of blood cell containing a horseshoe-shaped nucleus in their cytoplasm. They relate to the mononuclear phagocyte system and are much smaller than macrophages, which are in turn much smaller than multinucleated osteoclasts. Monocytes originate from bone marrow precursors [7]. Together with lymphocytes, monocytes were previously known as agranulocytes. Moreover, monocytes are defense cells of the second line after neutrophils in an innate immune system and are able to phagocytose foreign particles. They have the capability of differentiating into their own subsets and macrophages based on the necessity of the microenvironment. Additionally, monocytes produce cytokines and act as antigen-presenting cells (APCs) [8].

Monocytes differ from other autoimmune cells due to several reasons. First of all, they are capable of triggering, maintaining, and increasing the activity of inflammation in the synovial joints in RA. Moreover, chemokine receptors CCR2 (CD192) of CD14^+^ monocytes and CX3CR1 (fractalkine receptor, GPR13) of CD16^+^ monocytes interact with the corresponding ligands CCL2 (MCP-1) and CX3CL1 (fractalkine) released from FLSs, and this process contributes to the migration of monocytes from circulation and their recruitment into the RA synovium [9]. These chemoalkine receptors represent the differentiation markers of monocytes. A disintegrin and metalloproteinase domain-containing protein 10 and products of FLSs such as granulocyte–macrophage colony-stimulating factor (GM-CSF), TNF, and IL-1β promote monocyte migration. Next, monocytes are characterized by the increased expression of chemokine receptors and antigens on their cell surface [6]. Among them are CD14, CD16, toll-like receptors (TLRs), human leukocyte antigen DR (HLA-DR), and β1- and β2-integrins that are adhesion molecules. Moreover, they produce proinflammatory cytokines such as TNF, IL-1β, and IL-6. The differentiation of monocytes leads to the development of classical, intermediate, and non-classical monocyte subsets [10]. The intermediate monocytes are prevalent in RA, and they undergo differentiation into proinflammatory M1 macrophages. Proinflammatory macrophages are induced by TNF, GM-CSF, interferon (IFN)-γ, and lipopolysaccharide (LPS). M1 macrophages produce proinflammatory cytokines TNF, IL-1β, IL-6, and IL-12 and stimulate inflammation in the synovial joints [11]. Furthermore, classical monocytes expressing CD14^++^ and CD16^−^ have relatively greater potential to differentiate into osteoclasts and induce the erosion of the surrounding bones in the synovial joints in RA [12]. Osteoclastogenesis is the process by which osteoclasts are differentiated. Th17 cells trigger the formation of the receptor activator of the NF-κB (RANK) ligand (RANKL) by FLSs, which contributes to the differentiation of monocytes into osteoclasts [13]. Osteoclastogenesis is increased by cytokines such as TNF, IL-1β, and IL-6 [12,14].

The overview reveals the formation, classification, and specific markers of monocytes, their functioning as APCs and progenitors of macrophages and osteoclasts, the expression of chemokine receptors, and the production of cytokines in RA.

## 2. Monocytes in RA

### 2.1. Development of Monocytes

Monocytopoiesis, the process of monocyte production, originates from human stem cells, predominantly found in the bone marrow. Monocytes are part of the mononuclear phagocyte system and act as key innate immune cells in the organism. The process of monocytopoiesis occurs at a stage of embryonic development via definitive hematopoiesis. The ventral wall of the aorta is a site for the aorta gonad mesonephros emergence following three weeks of pregnancy in women and at the E-7 stage in mice. The aorta gonad mesonephros is the first location in the embryo where monocytes appear [15]. Hematopoietic stem cells differentiate into monoblasts under the influence of various cytokines, including GM-CSF and macrophage colony-stimulating factor (M-CSF). Hematopoietic stem cells undergo various stages of multipotent progenitor transformation into monocyte/macrophage and DC progenitors. These progenitors lose the ability to generate granulocytes and either give rise to “common monocyte progenitors” restricted to monocytes and their progeny or are directed to a common DC progenitor [15]. The subsequent stages involve further maturation and differentiation, giving rise to promonocytes and finally monocytes. These differentiated monocytes are released into the bloodstream, where they execute crucial functions such as phagocytosis, antigen presentation, and cytokine secretion, playing a pivotal role in immune surveillance and tissue homeostasis. Moreover, monocyte production happens in the liver, thymus, and spleen of the fetus. Since the late period of embryonic development, the formation of monocytes relocates to the bone marrow, which remains the main site for the monocyte production in adults and older people [16].

### 2.2. Human Monocyte Subsets and Their Role in RA

The expression of CD14 and CD16 markers on the cell surface of monocytes was used to identify and categorize the subsets of monocytes. According to the classification developed by Ziegler-Heitbrock et al., three types of monocyte subsets are presented in humans [7].

#### 2.2.1. Classical Monocytes

Primarily, there are classical monocytes, which have high expression of the CD14 marker and, more importantly, do not express the CD16 marker. Furthermore, they produce the cytokines TNF, IL-1β, IL-6, and IL-10 in high concentrations. Several forms of inflammatory stimulus are capable of eliciting the cytokine expression from monocytes. For instance, this process occurs as a consequence of the LPS stimulation and activation of the immune complex [17]. This type of monocyte undergoes differentiation into intermediate monocytes, inflammatory macrophages, and osteoclasts in the regions of inflammation. Classical monocytes function as phagocytic scavenger cells and promote synovial inflammation and the osteoclastic erosion of bones. CD16^−^, but not CD16^+^, monocyte subsets represent circulating osteoclast progenitors that differentiate into osteoclasts [12]. Consequently, classical monocytes are able to be the circulating predecessors of osteoclasts in erosive RA.

#### 2.2.2. Intermediate Monocytes

The second group refers to intermediate monocytes, which are characterized by the high expression of the CD14 marker and diminished expression of the CD16 marker. The number of these monocytes is highly upregulated in the peripheral blood and synovia in RA. They secrete TNF, IL-1β, and IL-6 in high quantities in the synovial joints in RA [18]. The differentiation of intermediate monocytes results in inflammatory monocyte subsets and proinflammatory M1 macrophages. They maintain the inflammation of the synovial joints and enhance the disease development. Additionally, intermediate monocytes lead to the direct activation of Th17 cells in the inflamed synovial joints and their expansion. The amount of these monocytes increases in the blood circulation over the course of the RA pathogenesis [19]. The enhanced level of classical and intermediate monocytes in an untreated RA patient is a prognostic factor for the decline or absence of susceptibility to methotrexate therapy [20].

#### 2.2.3. Non-Classical Monocytes

In addition, there are non-classical monocytes that have diminished expression of the CD14 marker and high expression of the CD16 marker. Non-classical monocytes preferentially associated with the endothelial wall engage in ”patrolling behavior” urgently in cases of tissue injury [21]. This process provides an early inflammatory response. Non-classical monocytes stimulate the adhesion of monocytes in the microvessels of joints. Resident anti-inflammatory M2 macrophages represent another product of monocyte differentiation and help to resolve inflammation. Among the monocyte subsets, the non-classical ones proliferate the least [10] and undergo senescence to a greater degree [22]. They are capable of acquiring a senescence-associated secreted phenotype (SASP) that transforms into a proinflammatory type and causes inflammation. Purchasing a phenotype occurs due to age-related chronic inflammation called inflammaging [23].

#### 2.2.4. Disease-Modifying Antirheumatic Drugs

The prevalence of circulating monocytes subsets, particularly the CD16 expressing subsets, is associated with RA progression and assists in estimating treatment effectiveness, for example, in the case of disease-modifying antirheumatic drugs (DMARDs). Commonly prescribed DMARDs include methotrexate, sulfasalazine, and hydroxychloroquine. Methotrexate inhibits dihydrofolate reductase, an enzyme that converts dihydrofolic acid into tetrahydrofolic acid. This enzyme, in turn, is a donor of one-carbon groups in the synthesis of purine nucleotides and thymidylate required for DNA synthesis. As a result, methotrexate inhibits DNA synthesis and repair, cell mitosis, and, to a lesser extent, affects RNA and protein synthesis [24]. Sulfasalazine dissociates into 5-aminosalicylic acid in the connective tissue of the intestinal wall, and 5-aminosalicylic acid promotes the anti-inflammatory properties of sulfasalazine and sulfapyridine [25]. Sulfapyridine is a competitive antagonist of para-aminobenzoic acid. This drug stops the synthesis of folate in the cells of microorganisms and causes antibacterial activity. Hydroxychloroquine seals lysosomal membranes and prevents the exit of lysosomal enzymes. Moreover, this drug disrupts DNA replication, RNA synthesis, and hemoglobin utilization by erythrocytic forms of plasmodium. Hydroxychloroquine weakens the activity of proteolytic enzymes, which are protease and collagenase. Additionally, this drug decreases the activity of leukocytes and the chemotaxis of lymphocytes. DMARDs help to prevent joint deformity and functional impairment [26].

### 2.3. Murine Monocyte Subsets in RA

Murine monocytes are subdivided into Ly6C^++^ CD43^+^, LY6C^++^ CD43^++^, and Ly6C^−^ types. The first one is an analogue of human classical monocytes. Another type resembles the intermediate monocytes. Both of these monocyte types trigger synovial inflammation under sterile conditions. The third type is an equivalent of non-classical monocytes and manages the progression and termination of sterile synovial inflammation [27]. In the beginning, Ly6C^−^ monocytes develop into proinflammatory M1 macrophages, which induce and maintain the inflammation in the synovial joints. Further, M1 macrophages are exposed to polarization and acquire an alternative phenotype, M2, that suppresses synovial inflammation [28].

### 2.4. Monocyte Markers

Monocytes are characterized by numerous antigen expression types on their cell surface, with some alterations in dependence on the monocyte subset. These antigens manage RA development [8].

#### 2.4.1. Markers of Double-Positive Monocytes/Macrophages

**Figure 1:**
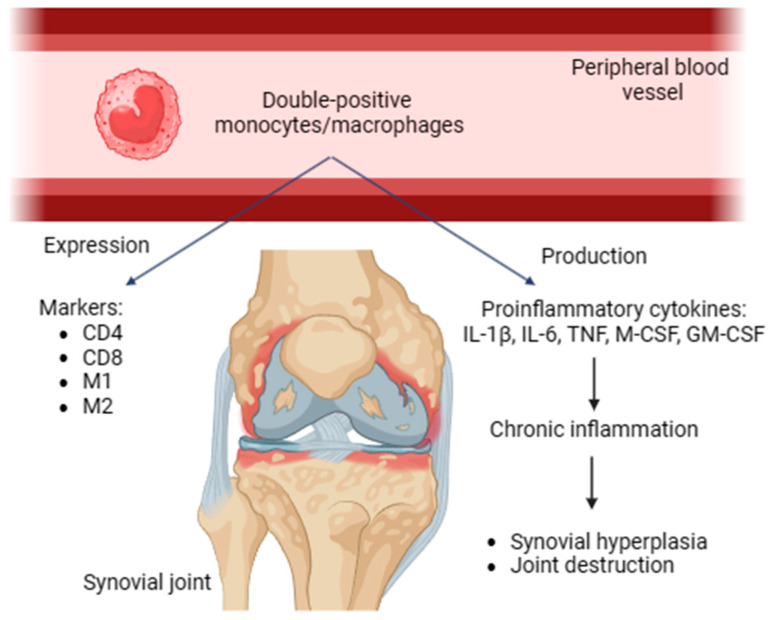
Double-positive monocytes/macrophages, expressed markers, proinflammatory (IL-1β, IL-6, TNF, M-CSF, and GM-CSF) cytokines, role in RA. CD, cluster of differentiation; IL, interleukin; TNF, tumor necrosis factor; GM-CSF, granulocyte–macrophage colony-stimulating factor.

Double-positive monocytes/macrophages were revealed in human and rat peripheral blood. These cells express both CD4 and CD8. In addition, they possess activated phenotypes expressing M1 and M2 markers, producing proinflammatory cytokines and contributing to chronic inflammation. Double-positive monocytes/macrophages may be transferred to the inflammation area and be involved in the immune response of Th1 type [29]. These cells promote synovial hyperplasia and facilitate joint damage in RA (Figure 1).

#### 2.4.2. Clusters of Differentiation

CD14 represents the LPS receptor, which is able to be linked to an LPS-binding protein. A CD14 interaction with an LPS–LPS binding protein complex initiates signaling pathways with the involvement of TLR-4. Further, TLR-4 triggers the formation and secretion of the proinflammatory chemokine IFN-γ inducible protein 10 and the cytokines TNF, IL-1β, and IL-6 [30]. CD16 designates the immunoglobulin (Ig) Fc-gamma receptor III (FcγRIII), which has a specific phagocytic activity. CD16^+^ monocytes, mostly the intermediate ones, are more abundant than non-classical monocytes in RA [19]. IgG-containing immune complexes, for instance, containing anti-citrullinated protein antibody (ACPA), provoke a higher level of TNF in the case of FcγRIII/CD16^+^ hyperexpression on CD14^++^ monocytes in RA. Magnetic microbeads for negative and positive selection with the use of anti-CD14 and anti-CD16 monoclonal antibodies allow separating the CD14 and CD16 markers [31]. CD56 is an adhesion molecule in neural cells. CD56^+^ monocytes mainly belong to classical monocyte subsets and spread in RA. Under the influence of LPS stimulation, CD14^bright^ CD56^+^ monocytes secrete greater amounts of TNF, IL-10, and IL-23. Anti-TNF therapy, for example, with the use of etanercept, diminishes the level of these cytokines [17]. Monocytes as well as myeloid series cells express the myeloid identification marker CD115 [32].

In addition to ACPAs, antibodies targeting various post-translational modifications such as carbamylation [33], acetylation, and malondialdehyde are found in RA. Approximately half of the ACPA clones in RA patients react with carbamylated antigens. Some antigens even show reactivity to acetylated peptides. The cross-reactivity may be due to similar electron density distributions at key recognized residues. Anti-malondialdehyde antibodies, although not cross-reactive with citrullinated antigens, can activate osteoclasts similar to ACPAs [34]. As a result, targeting antigens from different protein modifications trigger various pathways. Co-existing mechanisms and overlapping protein residues complicate the understanding of the key protein modifications for specific autoantibody effects. Various protein modifications can co-exist in the same tissues, cells, proteins, and even at the same peptide epitope. This co-existence explains the presence of autoantibodies targeting various post-translational modifications in RA and their similar functional effects. Colasanti T. et al. (2020) described the detection of antibodies targeting homocysteinylated alpha 1 antitrypsin in RA patients seropositive for the “classical” ACPA and RF. Alpha 1 antitrypsin is a protease inhibitor protein of the serpin superfamily and protects tissues from the effects of many enzymes secreted by inflammatory cells. Variation in the alpha 1 antitrypsin gene is associated with increased ACPA production, which leads to RA progression and joint destruction [35].

#### 2.4.3. HLA-DR and Co-Stimulatory Molecules

The HLA-DR marker refers to the group of MHC II (CD68) molecules. HLA-DR activates CD4^+^ cells via interaction with T cell receptors [36]. Intermediate monocytes produce a higher level of HLA-DR in comparison with other monocyte subsets in RA [37]. Furthermore, intermediate monocytes secrete a significant amount of TNF through Pam3Cys activation and binding to TLR2/TLR1. Classical monocytes increase the expression of HLA-DR over a period of low RA activity. Monocytes of the synovial joints are characterized by higher expression of HLA-DR than monocytes of the peripheral blood. Additionally, DCs demonstrate HLA-DR expression to a high degree and participate in antigen presentation to CD4^+^ T cells. HLA-DR interacts with an antigen and passes the first signal to the T cell receptor on a CD4^+^ T cell. Co-stimulatory molecules CD80 (B7-1) and CD86 (B7-2) are expressed on the monocyte surface and work as a second signal. To sum up, both signals stimulate CD4^+^ T-cells and enable adaptive autoimmune responses that happen with the involvement of Th1 and Th17 cells in RA. The expression of HLA-DR and CD80 is enhanced on the intermediate monocyte surface in the RA synovial joints, and this process contributes to the activation of IL-17^+^ and IFN-γ^+^ CD4^+^ T-cells under conditions of cocultivation [38]. Moreover, activated Th1 cells promote the expression of HLA-DR and CD80 on the surface of the monocytes in RA.

#### 2.4.4. Toll-like Receptors

TLRs are expressed on the surface of innate immune cells, for instance, monocytes, macrophages, DCs, and neutrophils. TLRs identify inflammatory tissues, microbial products, and cellular debris that remains after cell necrosis. Lipoteichoic acid and peptidoglycan recognition activate TLR2. LPS identification provokes TLR4 functioning [39]. Moreover, signal transduction through TLR2 and TLR4 is stimulated by molecules of heat shock protein 60, hyaluronic acid, and fibronectin products that are synthesized endogenously. The TLR level intensifies in resident and infiltrating cells in the RA synovium. Furthermore, TNF secretion is enhanced by increasing the TLR2 expression in CD16^+^ macrophages of blood and synovial origin in RA. The production of TLR2 grows with the assistance of classical and intermediate monocytes in the RA peripheral blood and synovial joints. All three monocyte subsets demonstrate a high TLR9 level on their surface independently of blood or synovial localization. TLR2 and TLR9 promote the early secretion of proinflammatory cytokines TNF and IL-1β [40]. Thereby, they induce inflammation that is maintained by their other products, such as the cytokine IL-6 and the chemokine macrophage inflammatory protein-1. Various levels of these proinflammatory factors are correlated with the involvement of TLR agonists. High expression of TLR3 and TLR4 is observed on fibroblasts in the RA synovial joints. In particular, continuous inflammation and joint damage cause this secretion [39].

Monocytes express the genes of type 1 interferons, especially in response to TLR signaling. Roelofs M.F. et al. (2009) described the co-expression of synovial TLR3/7 expression with IFN-α, IL-1β, and IL-18, but not with TNF, IL-12, or IL-17 [41]. The stimulation of TLR3/TLR7 on monocytes, monocyte-derived DCs, or synovial fibroblasts contributed to the secretion of type I IFN. However, no biologically active IL-1β or IL-18 could be revealed. Type I IFN-α increased the TLR3/7 mRNA expression, whereas IL-1β and IL-18 did not. In spite of the fact that the mRNA level of TLR4 remained unchanged, IFN-α enhanced the response to TLR4 agonists, a phenomenon that was clearly more marked in patients with RA. To conclude, type I IFNs are highly co-expressed with TLR3/TLR7 in RA synovium. They enhance TLR3/TLR7-mediated cytokine production and TLR4-mediated responses [41].

#### 2.4.5. β1- and β2-Integrins

The migration of circulating monocytes proceeds after their adhesion into the vascular endothelium. β1- and β2-integrins are expressed in high concentrations on monocytes in RA and participate in the adhesion of monocytes to activated endothelial cells. The β1 subfamily of integrins includes very late antigen-4 (α4β1) and -5 (α5β1) that bind to fibronectin and vascular cell adhesion molecule-1 in the endothelium and provoke monocyte adhesion [42]. β2-integrins take part in the formation of CD11_a,b,c_ (CD18) complexes in RA, which are mostly presented by CD11_b_ molecules and associated with the ligands in the endothelial lining. Moreover, intercellular adhesion molecule-1 (CD54), -2 (CD102), and -3 (CD50) cause monocyte adhesion to endothelial cells. Inflammatory monocytes/macrophages migrate into the synovial joints in RA and result in local inflammation [43].

#### 2.4.6. A Proliferation-Inducing Ligand

Two separate forms of a proliferation-inducing ligand (APRIL) are enhanced in the process of RA progression. The first form is a soluble one, which refers to the TNF superfamily and is secreted by myeloid cells, for instance, DCs, monocytes, macrophages, activated T cells, and B cells. Soluble APRIL operates as a proinflammatory cytokine and binds to receptors of two types. The first one is a transmembrane activator, calcium modulator and cyclophilin ligand interactor receptor (TACI), which is produced in B cells [44]. The second one is a B cell maturation antigen receptor (BCMA) that is secreted in plasma cells. The second form of APRIL is a receptor on the cell surface that is highly produced by the above-mentioned myeloid cells, including all the circulating monocyte subsets in RA. In the process of binding to TACI and BCMA, a surface form of APRIL functions as a ligand or receptor and provokes the formation and viability of B cells and plasma cells in RA. As a result, a B cell-mediated autoimmune response is provided [45].

#### 2.4.7. Sialic Acid Binding Ig-like Lectin

Siglec-1 is a representative of the sialic acid binding Ig-like lectin family. These proteins undergo upregulation by the circulating monocytes and macrophages in RA, and their concentration rises with the development of RA and increasing level of C-reactive protein, erythrocyte sedimentation rate, and IgM-rheumatoid factor. Singlec-1 operates as an autoantigen, takes part in a cell-to-cell contact of autoimmunity, and induces inflammation in RA [46].

#### 2.4.8. Transmembrane TNF

Transmembrane TNF is overexpressed on the monocyte surface in RA. Etanercept is a TNF inhibitor that binds to transmembrane TNF. Consequently, the anti-inflammatory cytokine IL-10 and soluble decoy receptors are produced. Thus, reverse signaling through transmembrane TNF occurs in monocytes and represents a prognostic factor for the pharmacological effect of anti-TNF agents in RA [47]. CD147 is an extracellular matrix metalloproteinase inducer, which promotes the production of matrix metalloproteinases (MMPs) by monocytes, for instance, MMP2, MMP3, and MMP9. MMPs provoke the dissolution of extracellular matrix, which leads to the destruction of synovium in RA. CD147 is hyperexpressed in CD14^+^ monocytes in the RA peripheral blood and synovial joints. Infliximab is a TNF inhibitor that is supposed to block the CD147 secretion on the surface of CD14^+^ monocytes in RA [48].

#### 2.4.9. Other Monocyte Markers

α-Enolase is an anti-citrullinated autoantigen for anti-citrullinated protein autoantibodies and is also expressed on the surface of monocytes/macrophages in RA. The main function of α-enolase is to initiate the secretion of proinflammatory cytokines, for example, TNF, IFN-γ, prostaglandin E2, and IL-1α/β [49]. Proteinase-activated receptor-2 is a member of the G protein receptor family and is produced by monocytes and T lymphocytes. The expression of proteinase-activated receptor-2 is directly proportional to the RA activity. In addition, the C-reactive protein and erythrocyte sedimentation rate concentrations increase over the course of the disease. Proteinase-activated receptor-2 initiates the production of the proinflammatory cytokine IL-6 [50]. Allograft inflammatory factor-1 is produced in the cytoplasm of monocytes/macrophages, lymphocytes, and synovial fibroblasts. Its function is to initiate and preserve inflammation in the synovial joints in RA. Hyperexpressed allograft inflammatory factor-1 participates in the proliferation of inflammatory macrophages and activated T lymphocytes in the RA synovium [51]. Moreover, 50–60% of CD14^+^ CD16^++^ non-classical monocytes have been shown to express the 6-sulfo LacNAc (SLAN) antigen, which is an O-linked glycosylated variant of P-selectin glycoprotein ligand-1 recognized by specific monoclonal antibodies, including macrophage-derived chemokine 8 and anti-D-dimers 1 and 2 [52,53]. In clinical studies, Hofer Th.P. et al. (2015) showed a fivefold depletion of SLAN-positive monocytes in patients with chronic inflammation [54]. SLAN is one of the monocyte markers stimulating the differentiation of non-classical monocytes into anti-inflammatory M2 macrophages, which, in turn, provide phagocytosis and resolution of inflammation.

Monocytes of the peripheral blood are progenitors of macrophages, and both of them mediate inflammation. Cardiovascular complications of RA are similar to synovitis and subclinical atherosclerosis, which have an inflammatory mechanism in the endothelium. Altered levels of the following receptor expression in circulating monocytes cause RA, accompanied by atherosclerotic complications, for instance, localized in a coronary artery. Specifically, low secretion of the scavenger receptor (CD36) is observed [55]. Moreover, a comorbidity is characterized by the high production of the calcium-sensing receptor and low-density lipoprotein-related receptor protein-1. The functional IL-25 receptor is secreted on the monocyte surface in RA and interacts with the cytokine IL-25, which is produced mostly by T cells. As a result, proinflammatory cytokine formation through the p38 mitogen-activated protein kinase (MAPK)-dependent Soc-3 pathway occurs. Additionally, this process signifies that IL-25 performs a negative control of monocyte-associated autoimmune disorders [56].

### 2.5. Cytokines

The cytokines participating in the RA pathways are subdivided into two types: proinflammatory and anti-inflammatory cytokines. The first type is responsible for the occurrence and development of synovial inflammation [57]. Conversely, the functioning of anti-inflammatory cytokines leads to a reduction in the inflammatory process. The cytokine environment of the RA synovium is formed by the cells of two groups. The first one includes resident synovial cells. The second group is represented by innate and adaptive autoimmunity cells. A higher secretion of cytokines is provided in the RA synovium to a greater extent by monocytes/macrophages and synovial fibroblasts than by T cells. The cytokines are involved in all the stages of RA development. Moreover, they unite the processes of immune regulation and inflammation that mediate the clinical symptoms and pathogenesis of RA [58].

#### 2.5.1. Proinflammatory Cytokines

IL-2 and IFN-γ are the main proinflammatory cytokines of Th1 cells. IL-2 stimulates the maturation and viability of T cells. IFN-γ initiates the functioning of HLA-DR. Moreover, IFN-γ localized in the synovial joints in RA triggers the development of inflammatory M1 macrophages [59]. IL-17 is a pleiotropic cytokine secreted by Th17 cells. This cytokine maintains the synovial inflammation. In addition, IL-17 participates in the activation of monocytes and promotes their migration. Furthermore, the Th17 cytokine stimulates the formation of cytokines by monocytes. There is a synergistic effect between IL-17 and TNF participating in the distribution of RA processes. Moreover, TNF increases osteoclastogenesis, as well as between IL-17 and IL-1β. These advantageous interactions lead to the activation of fibroblasts in the synovial joints, resulting in the production of proinflammatory cytokines and MMPs. Additionally, the Th17 cytokine provokes the erosion in the RA synovial joints. IL-17, in addition to TGF-β, IL-1β, and IL-23, increases osteoclastogenesis in RA [60].

TNF is mostly secreted by activated synovial FLSs and inflammatory macrophages in RA. Moreover, this cytokine is secreted by intermediate monocytes, T cells, and B cells. TNF participates in the distribution of RA processes. Moreover, TNF increases osteoclastogenesis in synovial inflammation. Infliximab is an effective anti-TNF agent that demonstrates the central role of TNF in RA development [61]. The B cell activator factor is a representative of the TNF family. Along with a soluble form of APRIL secreted by peripheral blood mononuclear cells, the B cell activator factor is able to be linked to B cells, resulting in B cell proliferation. Next, plasma cells are developed from autoreactive B cells and lead to the formation of RF and ACPA. Consequently, a B cell-mediated response occurs in RA, particularly in the early stages. Atacicept is an antagonist of APRIL and the B cell activator factor and is undergoing clinical trials at the moment [62].

In addition, FLSs release other proinflammatory cytokines in the RA synovial joints, for example, GM-CSF, IL-1β, IL-6, and IL-18. M1 macrophages ordinarily produce high concentrations of TNF, IL-1β, IL-6, and IL-12. With the secretion of TNF, Il-1β, and IL-6, monocytes stimulate an inflammatory process in the RA synovium. Adhesion molecules β1- and β2-integrins are expressed on the monocyte surface under the effect of proinflammatory cytokines. Intermediate monocytes are prevalent among the monocyte subsets that form the proinflammatory cytokines Il-1β, IL-6, and TNF in RA. Moreover, classical monocytes secrete the above-mentioned cytokines in accordance with the TLR agonists functioning [63]. The cytokine IL-18 is released by mononuclear cells in RA and is able to synergize with Il-1β, IL-12, and IL-15. Next, these molecules trigger the IFN-γ formation by activated synovial T cells and mediate the realization of the Th1 response. Moreover, IL-18 is capable of acting as a straight proinflammatory cytokine in RA and causing the secretion of TNF and IL-1β under the control of macrophages [64].

NF-kB signaling plays an important role in RA pathogenesis. This statement is confirmed by the NF-kB participation in the production of macrophage inflammatory protein-1a and the chemokine IL-8 [65]. Additionally, IL-15 stimulates neutrophils and delays the apoptosis of FLSs and endothelial cells over the period of synovial inflammation. Synovial fibroblasts are the main cells producing IL-23 in RA. Moreover, monocytes secrete IL-23 and initiate Th17 cell distribution and IL-17 expression in the RA synovial tissue. TNF, IL-1β, and IL-32 trigger inflammation in the murine synovium that is supposed to indicate their proinflammatory activity. IL-33 is a cytoplasmic representative of the IL-1 family and is expressed in activated monocytes and fibroblasts in the RA synovium. IL-33 undergoes emission from injured or necrotic cells and participates in the inflammation process. For instance, this protein controls the formation of mast cells. Furthermore, it manages the secretion of Th2 cytokines, such as IL-4, IL-5, and IL-13. Next, IL-33 promotes Th2-regulated pathological conditions [66].

#### 2.5.2. Anti-Inflammatory Cytokines

IL-1Ra is an IL-1 receptor antagonist formed by monocytes in RA. IL-1Ra and IL-10 are anti-inflammatory cytokines; therefore, their antagonistic action presupposes the decline and termination of inflammation. Nevertheless, IL-10 and TGF-β prevail among the products of Breg cells, Treg cells, and suppressor cells of myeloid origin. Il-4 and IL-10 are also secreted by Th2 cells. Moreover, Th2 cells enhance IL-1Ra expression. In addition, M2 macrophages secrete IL-10 and contribute to wound healing, tissue remodeling, and inflammation arrest. IL-10, as an anti-inflammatory cytokine, decreases inflammation. Therefore, a decrease in IL-10 production leads to the development of the inflammatory process and its transformation into a chronic form. IL-10 and M-CSF added to the cultivation medium promote monocyte survival, growth, and differentiation into macrophages [67]. Furthermore, monocytes express the cytokines IL-11, IL-19, IL-20, and IL-24. Additionally, the products of monocyte secretion are GM-CSF, G-CSF, and oncostatin M [68].

### 2.6. Chemokine Receptors

Chemokines binding to their receptors on the monocyte surface cause the return and migration of monocytes from the blood into the synovial tissue. CCR2 and CX3CR1 are chemokine receptors that are located on monocytes and trigger their chemotaxis. The first receptor is overexpressed on classical CD14^++^ CD16^−^ monocytes and binds to CCL2, which is a monocyte chemoattractant protein [69]. The second receptor undergoes high expression on predominantly intermediate CD14^++^ CD16^+^ monocytes and also non-classical CD14^+^ CD16^++^ monocytes in RA and interacts with CX3CL1. IL-1β cytokines and TNF induce the synovial fibroblasts to produce mainly CCL2, CX3CL1, and CCL20 in RA. CCL20, or macrophage inflammatory protein-3 alpha, is a different monocyte chemoattractant. Moreover, the c-Jun N-terminal kinase, extracellular signal-regulated kinase, and phosphatidylinositol 3′–kinase signaling pathways participate in the chemoattractant secretion by the Th17-associated cytokine IL-17. In addition, IL-17 has an indirect influence on monocyte migration [70].

Chemokine receptors undergo constitutive expression on peripheral blood and synovial monocytes/macrophages, mediate the monocyte chemotaxis, and maintain their level. The migration of peripheral blood monocytes to the synovial tissue during the inflammatory process promotes the increased concentration of chemokine receptors on their surface in RA [6]. Therefore, the regional inflammation and monocyte preservation in the synovium are defined by the overexpression of chemokine receptors on the RA monocytes. The increased expression of CCR1, CCR2, and CCR4 receptors is provided on peripheral blood monocytes, whereas synovial monocytes demonstrate high production of CCR3 and CCR5 in RA. Furthermore, the circulating monocytes secrete CCR9, which interacts with the CCL25 chemokine, or thymus-expressed chemokine. This binding results in monocytes’ development into macrophages in RA. The CCR7 chemokine receptor is secreted by not only B lymphocytes, CD4^+^ T lymphocytes, DCs, and FLSs but also circulating monocytes in RA. CCR7 binds to the CCL19 and CCL21 ligands that are characterized by hyperexpression in RA. The regulation of CCR7 and CCL19 is directly proportional to the RA progression. Moreover, the CCL19 concentration in plasma indicates the radiographic development of joint destruction. The DMARD therapy maintains the normal level of both components of the CCR7/CCL19 system [26].

## 3. Monocyte Functions in RA

The RA etiopathogenesis and its sensitivity to therapy are defined by genetic predisposition, environmental factors, infections, and the decline of autoimmune regulation. Frequent inflammation induces the development of autoimmune conditions. The pathogenesis of RA involves the activation and polarization of monocytes into proinflammatory M1 macrophages within the joint environment [71]. In the arthritic joint, monocytes are recruited by proinflammatory cytokines such as TNF, IL-1, and IL-6, which are produced by resident cells, including FLSs and macrophages. These cytokines promote the activation of monocytes through the binding of their receptors, leading to the upregulation of adhesion molecules and chemoattractant receptors on the monocyte surface. As a result, the monocytes adhere to the endothelium of blood vessels and migrate into the synovial tissue. Activated monocytes express increased levels of cell surface markers, such as CD80 and CD86, which facilitate interactions with T cells and promote antigen presentation [72]. M1-like macrophages are characterized by the production of proinflammatory cytokines and reactive oxygen species, which contribute to tissue damage and perpetuate inflammation in the joint. Furthermore, M1-like macrophages express high levels of surface markers, such as CD14 and CD16, which enhance their phagocytic and antigen-presenting capabilities.

### 3.1. Monocyte Interactions with Other Cells

Cells of innate and adaptive immunity work in cooperation to cause and maintain an inflammatory process in RA. The main cells taking part in this signaling are DCs, monocytes/macrophages, FLSs, CD4^+^ T cells (Th1, Th2, and Th17 cells), B cells, mast cells, and neutrophils. When immunoregulatory cells are not capable of attenuating acute inflammation, a chronic form of the disease develops. DCs, macrophages, and FLSs are activated at the beginning of RA pathogenesis [73].

Circulating monocytes are precursors of macrophages. Additionally, monocytes are able to operate as APCs and activate T cells. Moreover, they are classified as monocyte-derived DCs due to their capacity to behave as classical DCs [7]. Monocyte-derived DCs perform hyperexpression of IL-6 and IL-23. Tolerogenic monocyte-derived DCs are another type of DC that are capable of triggering the formation of Foxp3^+^ Treg cells in RA. Myeloid-derived suppressor cells (MDSCs), Breg cells, and Treg cells control and decrease the inflammatory process [74].

Among CD4^+^ T cells, the role of the Th1 and Th2 cells in the RA progression was initially revealed. The cytokine IL-12 mediates the participation of monocytes/macrophages in the polarization of CD4^+^ Th1 cells. Th2 cells and their cytokines mainly decrease Th1 activation. Intermediate monocytes prevail in the RA peripheral blood and synovium and are supposed to be the main monocyte subsets that modulate the Th17 cell functioning. Th17 cells were investigated as effectors in RA because of the ability of their pleiotropic cytokine IL-17 to synergize with TNF. IL-17A triggers the secretion of proinflammatory cytokines IL-1β, IL-6, and TNF in macrophages through the AP-1, NF-kB, and MAPK pathways [60]. IL-1β, IL-6, and IL-23 initiate the activation and control the polarization of Th17 cells. Activated T lymphocytes take part in cell-to-cell contact and trigger the secretion of TNF and IL-1β. Moreover, T lymphocytes release cytokine inhibitor secretory IL-1Ra in monocytes/macrophages [75].

Mast cells are a source of small-molecule inflammatory mediators. Neutrophils undergo chemotaxis into the RA synovial joints, mostly in the early period of inflammation, and release inflammatory mediators and enzymes. Over the period of joint effusion, the level of neutrophils predominates in the synovial fluid in comparison with the synovium [76].

A cytotoxic T-lymphocyte-associated antigen 4 (CTLA4) is a receptor for co-stimulatory molecules and is expressed on the surface of activated T cells [77]. CTLA4 binds with a high avidity to CD80 (B7-1) and CD86 (B7-2) on APCs. They are expressed on the APC surface and interact with T cell co-stimulatory receptor CD28 through signal 2 in order to activate the T cells. Moreover, CTLA4 promotes CD80 and CD86 transport to the CD28 receptor located on T cells for further activation of these co-stimulatory molecules [72]. CTLA4-Ig is a CTLA4 inhibitor and is registered as Abatacept. A blocking effect of Abatacept indicates a cell-to-cell contact mechanism of antigen presentation via T cell-monocyte/macrophage cooperation [77].

Moreover, a surface form of APRIL secreted by monocytes promotes cell communication with B cells and plasma cells that produce BCMA and TACI [44]. In addition, FLSs secrete soluble APRIL and, consequently, release BCMA. Furthermore, FLSs and synovial macrophages express CXCL16 and CCL20 chemokines, which interact with CCR6^+^ memory T cells. This communication, along with IL-15 cytokines, mainly contributes to CD4^+^ T cell migration and their involvement in the synovial tissue. Moreover, the enhanced transport of circulating monocytes into the RA synovial joints stimulates the participation of CXCR6^+^ T cells in the RA progression. Additionally, the CCL20 expression by activated monocytes occurs in the synovium of mice. Stimulated monocytes secrete CCL20, which contacts the CCR6 chemokine receptors, a product of Th17 cells. This interaction increases the CCL20 involvement in the synovial tissue. To sum up, cooperation between monocytes/macrophages, DCs, B cells, Th1, and Th17 cells is related to a positive feedback loop and has an effect on the continuation of inflammation in the synovial joints [60].

FLSs are the resident cells presenting in the intimal lining of the synovium along with resident macrophages, which are synoviocytes of type A. FLSs are highly proliferated in the synovial tissue, thus stimulating the hyperplasia of the synovium and damage to the joints. FLSs express RANKL and participate in the differentiation of osteoclasts and bone erosion [78]. CCL2 and CX3CL1 are FLS chemokines and behave as chemoattractants for monocytes/macrophages. Moreover, the corresponding MMPs eliminate the extracellular matrix. IL-6, IL-18, TNF, and GM-CSF, as the FLS cytokines, promote the stimulation of innate and adaptive immune cells [61].

Treg cells primarily decrease inflammation and regulate adaptive immunity. The Treg cells are determined as CD4^+^ CD25^+^ CD127^low^ FOXP3^+^ cells. There are two ways in which they cooperate with APCs and effector T cells. The first one is direct communication through cell-to-cell contact. The second way occurs with the help of anti-inflammatory cytokines, for instance, TGF-β, IL-4/IL-13, and IL-10. Furthermore, IL-10, as a potent anti-inflammatory cytokine, influences both the innate and adaptive immune cells [5]. CD8^+^ FoxP3^+^ Treg cells induced by anti-CD3 monoclonal antibodies decrease Th1 and Th17 cell activation through P38 phosphorylation inhibition and halt RA inflammation [74]. Monocytes promote the FoxP3 expression in CD8^+^ FoxP3^+^ Treg cells through direct cell contact. IL-10 decreases the Th17 cell amount and the IL-17 cytokine generation while simultaneously increasing the Foxp3^+^ Treg cell development. Thus, IL-10 modifies the ratio of Th17 cells and Treg cells in the CD4^+^ T cell population. Activated monocytes/macrophages are able to have both a positive and negative effect on the Treg cell phenotype and function, with the predominance of a negative one [74].

MDSCs are tumor-associated cells and take part in the formation of tumor-induced T regulatory cells and T cell anergy. There are two subsets of MDSCs. The first one is presented by monocytic MDSCs. Consequently, monocytes can be expanded and polarized towards MDSCs [13]. The second group includes granulocytic MDSCs. Both of these subsets suppress Th1 and Th17 cell proliferation. Moreover, they secrete IL-10 and contribute to the expansion of Treg cells in mice. To sum up, MDSCs possess immunoregulatory activity and repress RA synovial inflammation [4].

### 3.2. Monocyte Transport to the RA Synovial Tissue

Monocytes are the important innate effectors in the progression of RA. Circulating monocytes undergo chemotaxis and infiltration into the synovial tissue and stimulate synovial inflammation. Intermediate monocytes, and, to a high extent, the classical ones, are constantly involved in the local synovial inflammation development. This statement is confirmed by the increased presence of both of them in the RA synovium as well as the declining concentration of classical monocytes in the RA peripheral blood and a slightly enhanced number of intermediate monocytes there. M-CSF promotes the differentiation of classical monocytes into the intermediate subset, which indicates the heterogeneous character of the monocyte subsets and their possible polarization into their own other type [13]. The cooperation between CCL2/CCR2 and CX3CL1/CX3CR1 contributes to the migration of circulating monocytes and their functioning in the RA synovium. CCL2 is released by bone marrow mesenchymal stem cells and precursor cells. The CCR2/CCl2 complex on monocytes triggers their recruitment from the bone marrow into the bloodstream. The CX3CL1 production by FLSs is supported by disintegrin and metalloproteinase domain-containing protein 10 and disintegrin; therefore, they mediate the monocyte migration in RA [79].

### 3.3. Differentiation of Monocytes into Macrophages

In RA, monocytes are replaced by circulating monocytes, which constantly enter the synovium to support the local inflammation, although their part among the synovial macrophages is small. GM-CSF is released by activated synovial fibroblasts under the influence of IL-1β and TNF and provokes the survival of monocytes more than their polarization into macrophages [67]. The human stem cells from the fetal liver and erythro-myeloid predecessors supply the resident macrophages over the period of embryogenesis. However, circulating monocytes supply the recruited inflammatory macrophages when it is necessary, for example, in acute synovial inflammation. When the niche in the synovial tissue is free or unsaturated, macrophage development from circulating monocytes occurs. When there is no available niche, the macrophages are self-replenished by their own turnover. Intermediate monocyte subsets (CD14^++^ CD16^+^) are the prevailing monocytes in the RA synovial tissue, and they mainly differentiate into inflammatory M1 macrophages [30]. Synovial macrophages are located in the sub-lining and lining layers at the cartilage–pannus junction. The activated macrophages of the intimal lining release cytokines and provoke articular destruction. The rheumatoid factor autoantibodies and antigens form IgG-containing immune complexes, which stimulate the macrophages. Furthermore, innate immune cells, T cells, and fibroblasts secrete cytokines that have an impact on the stimulation of macrophages via cell-to-cell contact. The involved TLR-2 and TLR-4 activate macrophages and maintain their level in RA [39].

There are two major phenotypes of macrophages that are differentiated from monocytes in humans. The first is a proinflammatory type represented by classically activated M1 Mφ macrophages. They secrete high levels of IL-1 β, IL-6, IL-12, IL-23, TNF, and reactive oxygen species, which have the main impact on the continuation of the RA inflammation. The second type is anti-inflammatory and refers to alternatively activated Mφ M2 macrophages [80]. They, in turn, provide high concentrations of TGF-β, IL-1Ra, decoy IL-1RII, IL-10, and low IL-12 content. The Mφ M2 macrophage functions are anti-inflammatory activity, tissue remodeling, and wound healing. Both the proinflammatory and anti-inflammatory macrophage phenotypes take part in inflammation regulation. Their differentiation from monocytes with the use of the GM-CSF and M-CSF induction cytokines leads to Mø M1 and Mø2 M2 formation, correspondingly [68]. Moreover, activated Mφ M2 macrophages have three subsets according to their stimulating cytokines. There are three M2 forms. The first one is an alternative M2 form called M2a and triggered by IL-4 and IL-13. The second one is M2b, which is initiated by the impact of the IgG-containing immune complex and the TLR agonists or IL-1R. The third M2 form is M2c, which is induced by IL-10 and glucocorticoid hormones [60].

Th1 cells, Th2 cells, and Th17 cells, along with their associated cytokines, play a crucial role in the polarization, recruitment, activation, and differentiation of monocytes/macrophages [59]. The activation and differentiation of M1 macrophages are driven by the Th1 cytokine IFN-γ in the presence of LPS and TNF. On the other hand, M2 macrophages are driven by Th2 cytokines such as IL-4, IL-10, and IL-13. CXCL5, CXCL8, CXCL9, CXCL10, and CXCL13 are typical chemokines expressed by M1 macrophages. Notably, in vitro studies have demonstrated that M-CSF polarized M2 macrophages can produce proinflammatory cytokines in response to an IgG immune complex containing ACPA. This finding is also relevant in the context of RA since the synovium exhibits elevated levels of M-CSF [67]. Furthermore, activin A, found in RA synovial fluid, induces increased expression of MMP12 (a proinflammatory polarization marker) on monocytes/macrophages, polarizing them into proinflammatory M1 phenotypes. Given these findings, activin A levels may serve as a potential biomarker for various therapies.

The overexpression of the Silent Information Regulator-2 homolog, SIRT1, on monocytes has been shown to downregulate PU.1 phosphorylation, thereby inhibiting their differentiation into macrophages. SIRT1 overexpression can inhibit the production of proinflammatory cytokines by blocking the NF-kB pathway in RA patients. Consequently, SIRT1 has emerged as a significant therapeutic target of interest due to its role in regulating the inflammatory process in RA [81]. It is worth mentioning that monocytes/macrophages secrete the cytosolic phospholipase A2a enzyme, which plays a crucial role in generating prostaglandin E2 from cell membrane phospholipids [82]. Prostaglandin E2, in turn, contributes to the induction and maintenance of RA inflammation. In addition, under the influence of LPS, TNF, and IL-1β, osteoblastic stromal cells produce cytosolic phospholipase A2a, ultimately leading to the generation of prostaglandin E2. This process has been associated with the promotion of inflammatory bone resorption in RA.

### 3.4. Monocytes Are Osteoclast Predecessors

Periarticular osteopenia and bone erosion adjacent to the pannus formation of synovial joints are two of the most destructive events observed in RA with high disease activity. The main culprits responsible for this erosion are osteoclasts, the primary bone resorbing cells. Osteoclasts originate from circulating monocytes and resident macrophages, which continuously migrate into the inflamed synovium and differentiate into osteoclasts, particularly in active disease and bone erosion. Recent research has shown that monocytes expressing CD14^+^ and lacking CD16^−^ are the main precursors of osteoclast progenitors, as opposed to CD16^+^ subsets [12]. These monocytes upregulate the RANK on their surface and interact with RANKL, which is predominantly expressed by FLSs, osteoblasts, and activated T cells. Th17 cells also play a significant role in promoting osteoclastogenesis as they stimulate the expression of RANKL, which preferably interacts with the CD14^+^ monocytes expressing the surface marker CCR6, characteristic of Th17 cells [83]. Furthermore, the production of IL-17, along with TNF, IL-1β, and IL-6, can amplify osteoclastogenesis. Additionally, the mesenchymal stromal cells secreted by FLSs, when synergized with RANKL, also aid in promoting osteoclastogenesis [56].

The mature activated osteoclasts release hydrochloric acid near their ruffled border to dissolve the calcium from the bone matrix and also produce MMPs and cathepsin K, which break down the remaining bone matrix, resulting in bone erosion adjacent to the joints [48]. RA monocytes exhibit increased expression of the co-stimulatory osteoclast-associated receptor, which, upon interaction with the collagen type II and collagen type I ligands in the inflamed synovial articular cartilage, further promotes the formation of osteoclasts. However, the osteoblasts’ soluble decoy receptor osteoprotegerin negatively affects osteoclastogenesis and disrupts the osteoprotegerin/RANK ratio, consequently favoring osteogenesis [12]. The activated T cells expressing the inducible costimulator, when interacting with the inducible costimulator ligand (CD275) expressed by monocyte-derived osteoclast-like cells, obstruct their differentiation into osteoclasts. This interaction suppresses the expression of tartrate-resistant acid phosphatase, the nuclear factor of activated T cells, and the osteoclast-associated receptors in monocyte-derived osteoclast-like cells during their maturation into osteoclasts. Thus, the CD275-inducible costimulator system directly interferes with osteoclastogenesis [84].

Clinical trials in RA focused on the monocytes that are of interest. The Chinese government approved sinomenine as an anti-inflammation drug for RA treatment. Liu W. et al. (2018) screened the various secretory cytokines in both LPS-induced and sinomenine-treated RAW264.7 cells, followed by an estimation of the sinomenine ability to modulate the cytokine secretion in a cell model, a collagen-induced arthritis mouse model, and RA patients [2]. The results demonstrated that sinomenine regulated the IL-6, GM-CSF, IL-12 p40, IL-1α, TNF, IL-1β, CXCL1, Eotaxin-2, IL-10, M-CSF, RANTES, and CCL2 secretion in vivo and in vitro and reduced the RA activity and the 28-joint disease activity score in a clinical setting. Moreover, sinomenine attenuated the CD11b^+^F4/80^+^CD64^+^ resident macrophages in the synovial tissue, the CD11b^+^Ly6C^+^CD43^+^ macrophages in the spleen, and draining the lymph nodes in collagen-induced arthritic mice. The percentage of CD14^+^CD16^+^ peripheral blood mononuclear cells was reduced by sinomenine in the RA patients. In conclusion, sinomenine controls the secretion of multiple inflammatory cytokines and monocyte/macrophage subsets, thus decreasing RA development. Along with methotrexate, sinomenine could be an alternative as a cost-effective anti-inflammatory agent for treating RA [2].

## 4. Discussion

The innate and adaptive autoimmunity cells within the interconnected network drive the continuous progression of RA pathogenesis. Numerous studies conducted to date, as well as targeted therapies, have demonstrated that FLSs, macrophages, Th1 cells, and B cells play a pivotal role in initiating and advancing synovial inflammation [85]. Recently, Th17 cells have been implicated as the primary drivers of immune cell activation and cytokine production [60]. Furthermore, they are primarily responsible for inducing RANKL production by FLSs and osteoblasts, thereby promoting osteoclastogenesis [78]. Presently, the data suggest that monocyte activation occurs within the circulation of individuals with RA. These activated monocytes respond to chemotactic ligands such as CCL2 and CX3CL1, migrating from the circulation into the RA synovium and releasing proinflammatory cytokines [83]. Thus, their involvement in perpetuating synovial inflammation in RA cannot be disregarded. Additionally, they function as APCs, activating other autoimmune cells and stimulating the production of their respective cytokines. Evidently, they partake in a positive feedback loop with the other key cells involved in RA pathogenesis. The increased expression of various antigens on the monocyte membrane surface can be a potential target for therapy in RA. Evaluating the levels of total monocytes and their subsets in the peripheral blood of the RA patients, both before and after therapy, can provide rheumatologists with guidance in determining the preference for treatment with DMARDs or biologic agents [26]. Elevated levels of monocytes in the peripheral blood of RA patients may serve as an additional biomarker for high disease activity, as evidenced by the correlation with the disease activity score-28 scores and the serum levels of C-reactive protein and the erythrocyte sedimentation rate.

The display of a heterogeneous character by the monocytes enables the differentiation into subsets based on the microenvironment or inflammatory stages and, more importantly, the ability to transform into intermediate monocytes. This emphasizes the high plasticity of the monocytes in the pathogenesis of RA. Inflammatory M1 macrophages, which are crucial players in the development of RA, are primarily derived from circulating monocytes, specifically intermediate monocytes [2]. This raises the question of which chemical events, signals, or specific factors, apart from the arthritic joint environment, trigger the conversion of CD14 monocytes to CD14^++^ CD16^+^ intermediate inflammatory macrophages [86]. Focusing on these studies would not only benefit RA patients but also shed light on other diseases where CD14^++^ CD16^+^ macrophages play a significant role, such as atherosclerosis. Classical monocytes expressing CD14^++^ and CD16^−^ are responsible for the bone erosion observed in erosive RA [87]. Osteoclastogenesis, the formation of osteoclasts, is influenced by the presence of FLSs and Th17 cells. FLSs produce RANKL and M-CSF to promote osteoclastogenesis [67]. Additionally, IL-17, produced by Th17 cells, induces the production of RANKL by FLSs. Furthermore, IL-17, along with TNF, IL-1β, and IL-6, amplifies osteoclastogenesis [88]. However, it is equally important to consider the availability of classical monocytes in the RA synovium or microcirculation near RA synovial joints in erosive rheumatoid arthritis as they have been shown to be precursor cells capable of differentiating into osteoclasts.

## 5. Conclusions

Recent discussions have highlighted the significant advancements in our understanding of how innate immune cells and signaling play a crucial role in driving the pathogenesis of RA. This complexity of the disease underscores the importance of targeting the innate immune system and its components for potential therapeutic interventions that can reduce the incidence and enhance the quality of life for RA patients. To further explore this, additional research is needed to investigate how the heterogeneity and plasticity of monocytes, the antigenic expressions on their surface, the differentiation into macrophages and osteoclasts, the migration into the synovium, and the role of their cytokines in RA influence their function in RA development. Moreover, studying how macrophage polarization in inflamed joints changes during the course of RA can provide valuable insights into the intricate and pivotal role of these cells in the disease process.

## Data Availability

Data available from the authors.

## References

[1] Cheng Q., Wu H., Du Y. (2021). The roles of small-molecule inflammatory mediators in rheumatoid arthritis. Scand. J. Immunol..

[2] Liu W., Zhang Y., Zhu W., Ma C., Ruan J., Long H., Wang Y. (2018). Sinomenine Inhibits the Progression of Rheumatoid Arthritis by Regulating the Secretion of Inflammatory Cytokines and Monocyte/Macrophage Subsets. Front. Immunol..

[3] Balchin C., Tan A.L., Golding J., Bissell L.-A., Wilson O.J., McKenna J., Stavropoulos-Kalinoglou A. (2022). Acute effects of exercise on pain symptoms, clinical inflammatory markers and inflammatory cytokines in people with rheumatoid arthritis: A systematic literature review. Ther. Adv. Musculoskelet. Dis..

[4] Morell M., Varela N., Marañón C. (2017). Myeloid Populations in Systemic Autoimmune Diseases. Clin. Rev. Allergy Immunol..

[5] Mishra S., Srinivasan S., Ma C., Zhang N. (2021). CD8+ Regulatory T Cell—A Mystery to Be Revealed. Front. Immunol..

[6] Choi J., Selmi C., Leung P.S.C., Kenny T.P., Roskams T., Gershwin M.E. (2016). Chemokine and chemokine receptors in autoimmunity: The case of primary biliary cholangitis. Expert Rev. Clin. Immunol..

[7] Ziegler-Heitbrock L., Ancuta P., Crowe S., Dalod M., Grau V., Hart D.N., Leenen P.J.M., Liu Y.-J., MacPherson G., Randolph G.J. (2010). Nomenclature of monocytes and dendritic cells in blood. Blood.

[8] Liu E.G., Yin X., Swaminathan A., Eisenbarth S.C. (2021). Antigen-Presenting Cells in Food Tolerance and Allergy. Front. Immunol..

[9] Li L., Huang L., Sung S.-S.J., Vergis A.L., Rosin D.L., Rose C.E., Lobo P.I., Okusa M.D. (2008). The chemokine receptors CCR2 and CX3CR1 mediate monocyte/macrophage trafficking in kidney ischemia-reperfusion injury. Kidney Int..

[10] Wolf A.A., Yáñez A., Barman P.K., Goodridge H.S. (2019). The Ontogeny of Monocyte Subsets. Front. Immunol..

[11] Mushenkova N.V., Nikiforov N.G., Shakhpazyan N.K., Orekhova V.A., Sadykhov N.K., Orekhov A.N. (2022). Phenotype Diversity of Macrophages in Osteoarthritis: Implications for Development of Macrophage Modulating Therapies. Int. J. Mol. Sci..

[12] Komano Y., Nanki T., Hayashida K., Taniguchi K., Miyasaka N. (2006). Identification of a human peripheral blood monocyte subset that differentiates into osteoclasts. Arthritis Res. Ther..

[13] Chen S., Guo C., Wang R., Feng Z., Liu Z., Wu L., Zhao D., Zheng S., Chen F., Zhang D. (2021). Monocytic MDSCs skew Th17 cells toward a pro-osteoclastogenic phenotype and potentiate bone erosion in rheumatoid arthritis. Rheumatology.

[14] Luukkonen J., Huhtakangas J., Palosaari S., Tuukkanen J., Vuolteenaho O., Lehenkari P. (2022). Preliminary Report: Osteoarthritis and Rheumatoid Arthritis Synovial Fluid Increased Osteoclastogenesis In Vitro by Monocyte Differentiation Pathway Regulating Cytokines. Mediators Inflamm..

[15] De Kleer I., Willems F., Lambrecht B., Goriely S. (2014). Ontogeny of myeloid cells. Front. Immunol..

[16] Weaver L.K., Chu N., Behrens E.M. (2016). TLR9-mediated inflammation drives a Ccr2-independent peripheral monocytosis through enhanced extramedullary monocytopoiesis. Proc. Natl. Acad. Sci. USA.

[17] Krasselt M., Baerwald C., Wagner U., Rossol M. (2013). CD56+ monocytes have a dysregulated cytokine response to lipopolysaccharide and accumulate in rheumatoid arthritis and immunosenescence. Arthritis Res. Ther..

[18] Sørensen A.S., Andersen M.N., Juul-Madsen K., Broksø A.D., Skejø C., Schmidt H., Vorup-Jensen T., Kragstrup T.W. (2022). Tumor necrosis factor alpha neutralization attenuates immune checkpoint inhibitor-induced activation of intermediate monocytes in synovial fluid mononuclear cells from patients with inflammatory arthritis. Arthritis Res. Ther..

[19] Tsukamoto M., Seta N., Yoshimoto K., Suzuki K., Yamaoka K., Takeuchi T. (2017). CD14brightCD16+ intermediate monocytes are induced by interleukin-10 and positively correlate with disease activity in rheumatoid arthritis. Arthritis Res. Ther..

[20] Chara L., Sánchez-Atrio A., Pérez A., Cuende E., Albarrán F., Turrión A., Chevarria J., del Barco A.A., Sánchez M.A., Monserrat J. (2015). The number of circulating monocytes as biomarkers of the clinical response to methotrexate in untreated patients with rheumatoid arthritis. J. Transl. Med..

[21] Buscher K., Marcovecchio P., Hedrick C.C., Ley K. (2017). Patrolling Mechanics of Non-Classical Monocytes in Vascular Inflammation. Front. Cardiovasc. Med..

[22] Ong S.-M., Hadadi E., Dang T.-M., Yeap W.-H., Tan C.T.-Y., Ng T.-P., Larbi A., Wong S.-C. (2018). The pro-inflammatory phenotype of the human non-classical monocyte subset is attributed to senescence. Cell Death Dis..

[23] Rodríguez-Vargas G.-S., Santos-Moreno P., Rubio-Rubio J.-A., Bautista-Niño P.-K., Echeverri D., Gutiérrez-Castañeda L.-D., Sierra-Matamoros F., Navarrete S., Aparicio A., Saenz L. (2022). Vascular Age, Metabolic Panel, Cardiovascular Risk and Inflammaging in Patients with Rheumatoid Arthritis Compared with Patients with Osteoarthritis. Front. Cardiovasc. Med..

[24] Frouin I., Prosperi E., Denegri M., Negri C., Donzelli M., Rossi L., Riva F., Stefanini M., Scovassi A.I. (2001). Different effects of methotrexate on DNA mismatch repair proficient and deficient cells. Eur. J. Cancer.

[25] Greenstein R.J., Su L., Shahidi A., Brown S.T. (2007). On the action of 5-amino-salicylic acid and sulfapyridine on M. avium including subspecies paratuberculosis. PLoS ONE.

[26] Deng G., Chen X., Shao L., Wu Q., Wang S. (2023). Effectiveness and safety of 99Tc-methylene diphosphonate as a disease-modifying anti-rheumatic drug (DMARD) in combination with conventional synthetic (cs) DMARDs in the treatment of rheumatoid arthritis: A systematic review and meta-analysis of 34 randomized controlled trials. Heliyon.

[27] Ammari M., Presumey J., Ponsolles C., Roussignol G., Roubert C., Escriou V., Toupet K., Mausset-Bonnefont A.-L., Cren M., Robin M. (2018). Delivery of miR-146a to Ly6Chigh Monocytes Inhibits Pathogenic Bone Erosion in Inflammatory Arthritis. Theranostics.

[28] Ledesma-Colunga M.G., Baschant U., Weidner H., Alves T.C., Mirtschink P., Hofbauer L.C., Rauner M. (2023). Transferrin receptor 2 deficiency promotes macrophage polarization and inflammatory arthritis. Redox Biol..

[29] Roberts C.A., Dickinson A.K., Taams L.S. (2015). The Interplay Between Monocytes/Macrophages and CD4(+) T Cell Subsets in Rheumatoid Arthritis. Front. Immunol..

[30] Zamani F., Shahneh F.Z., Aghebati-Maleki L., Baradaran B. (2013). Induction of CD14 Expression and Differentiation to Monocytes or Mature Macrophages in Promyelocytic Cell Lines: New Approach. Adv. Pharm. Bull..

[31] Rodrigues C.P., Ferreira A.C.F., Pinho M.P., de Moraes C.J., Bergami-Santos P.C., Barbuto J.A.M. (2016). Tolerogenic IDO(+) Dendritic Cells Are Induced by PD-1-Expressing Mast Cells. Front. Immunol..

[32] Forget A., Gianni-Barrera R., Uccelli A., Sarem M., Kohler E., Fogli B., Muraro M.G., Bichet S., Aumann K., Banfi A. (2019). Mechanically Defined Microenvironment Promotes Stabilization of Microvasculature, Which Correlates with the Enrichment of a Novel Piezo-1+ Population of Circulating CD11b+ /CD115+ Monocytes. Adv. Mater..

[33] Spinelli F.R., Pecani A., Conti F., Mancini R., Alessandri C., Valesini G. (2016). Post-translational modifications in rheumatoid arthritis and atherosclerosis: Focus on citrullination and carbamylation. J. Int. Med. Res..

[34] Sakuraba K., Krishnamurthy A., Sun J., Zheng X., Xu C., Peng B., Engström M., Jakobsson P.-J., Wermeling F., Catrina S. (2022). Autoantibodies targeting malondialdehyde-modifications in rheumatoid arthritis regulate osteoclasts via inducing glycolysis and lipid biosynthesis. J. Autoimmun..

[35] Colasanti T., Sabatinelli D., Mancone C., Giorgi A., Pecani A., Spinelli F.R., Giamberardino A.D., Navarini L., Speziali M., Vomero M. (2020). Homocysteinylated alpha 1 antitrypsin as an antigenic target of autoantibodies in seronegative rheumatoid arthritis patients. J. Autoimmun..

[36] Costantino C.M., Ploegh H.L., Hafler D.A. (2009). Cathepsin S regulates class II MHC processing in human CD4+ HLA-DR+ T cells. J. Immunol..

[37] Wahyurini D., Wibowo H. (2023). Monocytic HLA-DR expression in type 2 diabetes mellitus: Impact on disease susceptibility. World J. Adv. Res. Rev..

[38] Iwamoto N., Kawakami A. (2022). The monocyte-to-osteoclast transition in rheumatoid arthritis: Recent findings. Front. Immunol..

[39] Hwang S., Sung D.K., Kim Y.E., Yang M., Ahn S.Y., Sung S.I., Chang Y.S. (2023). Mesenchymal Stromal Cells Primed by Toll-like Receptors 3 and 4 Enhanced Anti-Inflammatory Effects against LPS-Induced Macrophages via Extracellular Vesicles. Int. J. Mol. Sci..

[40] Wu X.-Y., Li K.-T., Yang H.-X., Yang B., Lu X., Zhao L.-D., Fei Y.-Y., Chen H., Wang L., Li J. (2020). Complement C1q synergizes with PTX3 in promoting NLRP3 inflammasome over-activation and pyroptosis in rheumatoid arthritis. J. Autoimmun..

[41] Roelofs M.F., Wenink M.H., Brentano F., Abdollahi-Roodsaz S., Oppers-Walgreen B., Barrera P., van Riel P.L.C.M., Joosten L.A.B., Kyburz D., van den Berg W.B. (2009). Type I interferons might form the link between Toll-like receptor (TLR) 3/7 and TLR4-mediated synovial inflammation in rheumatoid arthritis (RA). Ann. Rheum. Dis..

[42] Park J.-Y., Park H.-M., Kim S., Jeon K.-B., Lim C.-M., Hong J.T., Yoon D.-Y. (2023). Human IL-32θA94V mutant attenuates monocyte-endothelial adhesion by suppressing the expression of ICAM-1 and VCAM-1 via binding to cell surface receptor integrin αVβ3 and αVβ6 in TNF-α-stimulated HUVECs. Front. Immunol..

[43] Luo Z., The E., Zhang P., Zhai Y., Yao Q., Ao L., Zeng Q., Fullerton D.A., Meng X. (2022). Monocytes augment inflammatory responses in human aortic valve interstitial cells via β2-integrin/ICAM-1-mediated signaling. Inflamm. Res..

[44] Mantchev G.T., Cortesão C.S., Rebrovich M., Cascalho M., Bram R.J. (2007). TACI is required for efficient plasma cell differentiation in response to T-independent type 2 antigens. J. Immunol..

[45] Nagatani K., Itoh K., Nakajima K., Kuroki H., Katsuragawa Y., Mochizuki M., Aotsuka S., Mimori A. (2007). Rheumatoid arthritis fibroblast-like synoviocytes express BCMA and are stimulated by APRIL. Arthritis Rheum..

[46] Xiong Y.-S., Cheng Y., Lin Q.-S., Wu A.-L., Yu J., Li C., Sun Y., Zhong R.-Q., Wu L.-J. (2014). Increased expression of Siglec-1 on peripheral blood monocytes and its role in mononuclear cell reactivity to autoantigen in rheumatoid arthritis. Rheumatology.

[47] Meusch U., Krasselt M., Rossol M., Baerwald C., Klingner M., Wagner U. (2015). In vitro response pattern of monocytes after tmTNF reverse signaling predicts response to anti-TNF therapy in rheumatoid arthritis. J. Transl. Med..

[48] Huang J., Xie B., Li Q., Xie X., Zhu S., Wang M., Peng W., Gu J. (2013). Infliximab reduces CD147, MMP-3, and MMP-9 expression in peripheral blood monocytes in patients with active rheumatoid arthritis. Eur. J. Pharmacol..

[49] Ebrahimi-Rad M., Khatami S., Akhbari H., Mahmoudzadeh-Niknam H., Valadbeigi S., Mahmoudi M., Jamshidi A., Riazi-Rad F., Saghiri R. (2020). Evaluation of autoantibodies against vimentin and α-enolase in rheumatoid arthritis patients. Reumatologia.

[50] Crilly A., Burns E., Nickdel M.B., Lockhart J.C., Perry M.E., Ferrell P.W., Baxter D., Dale J., Dunning L., Wilson H. (2012). PAR(2) expression in peripheral blood monocytes of patients with rheumatoid arthritis. Ann. Rheum. Dis..

[51] Piotrowska K., Słuczanowska-Głabowska S., Kurzawski M., Dziedziejko V., Kopytko P., Paczkowska E., Rogińska D., Safranow K., Machaliński B., Pawlik A. (2020). Over-Expression of Allograft Inflammatory Factor-1 (AIF-1) in Patients with Rheumatoid Arthritis. Biomolecules.

[52] Hofer T.P., van de Loosdrecht A.A., Stahl-Hennig C., Cassatella M.A., Ziegler-Heitbrock L. (2019). 6-Sulfo LacNAc (Slan) as a Marker for Non-classical Monocytes. Front. Immunol..

[53] Tamassia N., Bianchetto-Aguilera F., Gasperini S., Grimaldi A., Montaldo C., Calzetti F., Gardiman E., Signoretto I., Castellucci M., Barnaba V. (2023). The slan antigen identifies the prototypical non-classical CD16+-monocytes in human blood. Front. Immunol..

[54] Hofer T.P., Zawada A.M., Frankenberger M., Skokann K., Satzl A.A., Gesierich W., Schuberth M., Levin J., Danek A., Rotter B. (2015). slan-defined subsets of CD16-positive monocytes: Impact of granulomatous inflammation and M-CSF receptor mutation. Blood.

[55] Gómez-Bañuelos E., Martín-Márquez B.T., Martínez-García E.A., Figueroa-Sanchez M., Nuñez-Atahualpa L., Rocha-Muñoz A.D., Sánchez-Hernández P.E., Navarro-Hernandez R.E., Madrigal-Ruiz P.M., Saldaña-Millan A.A. (2014). Low levels of CD36 in peripheral blood monocytes in subclinical atherosclerosis in rheumatoid arthritis: A cross-sectional study in a Mexican population. Biomed. Res. Int..

[56] Min H.K., Won J.-Y., Kim B.-M., Lee K.-A., Lee S.-J., Lee S.-H., Kim H.-R., Kim K.-W. (2020). Interleukin (IL)-25 suppresses IL-22-induced osteoclastogenesis in rheumatoid arthritis via STAT3 and p38 MAPK/IκBα pathway. Arthritis Res. Ther..

[57] Pan S., Wu S., Wei Y., Liu J., Zhou C., Chen T., Zhu J., Tan W., Huang C., Feng S. (2024). Exploring the causal relationship between inflammatory cytokines and inflammatory arthritis: A Mendelian randomization study. Cytokine.

[58] Filali S., Noack M., Géloën A., Pirot F., Miossec P. (2023). Effects of pro-inflammatory cytokines and cell interactions on cell area and cytoskeleton of rheumatoid arthritis synoviocytes and immune cells. Eur. J. Cell Biol..

[59] Gloyer L., Golumba-Nagy V., Meyer A., Yan S., Schiller J., Breuninger M., Jochimsen D., Kofler D.M. (2022). Adenosine receptor A2a blockade by caffeine increases IFN-gamma production in Th1 cells from patients with rheumatoid arthritis. Scand. J. Rheumatol..

[60] Park J.-S., Kim N.-R., Lim M.-A., Kim S.-M., Hwang S.-H., Jung K.-A., Choi J.W., Park S.-H., Cho M.-L. (2018). Deficiency of IL-1 receptor antagonist suppresses IL-10-producing B cells in autoimmune arthritis in an IL-17/Th17-dependent manner. Immunol. Lett..

[61] Yan J., Yao L., Tan Y., Wang Y. (2023). The protective effects of Phoenixin-20 in tumor necrosis factor α (TNF-α)-induced cell senescence of rheumatoid arthritis fibroblast-like synoviocytes (FLS). Aging.

[62] Shabgah A.G., Shariati-Sarabi Z., Tavakkol-Afshari J., Ghasemi A., Ghoryani M., Mohammadi M. (2020). A significant decrease of BAFF, APRIL, and BAFF receptors following mesenchymal stem cell transplantation in patients with refractory rheumatoid arthritis. Gene.

[63] Okamato Y., Ghosh T., Okamoto T., Schuyler R.P., Seifert J., Charry L.L., Visser A., Feser M., Fleischer C., Pedrick C. (2021). Subjects at-risk for future development of rheumatoid arthritis demonstrate a PAD4-and TLR-dependent enhanced histone H3 citrullination and proinflammatory cytokine production in CD14hi monocytes. J. Autoimmun..

[64] Stangl H., Krammetsvogl A., Lesiak M., Wolff C., Straub R.H. (2020). MHC/class-II-positive cells inhibit corticosterone of adrenal gland cells in experimental arthritis: A role for IL-1β, IL-18, and the inflammasome. Sci. Rep..

[65] Murphy E.P., Crean D. (2015). Molecular Interactions between NR4A Orphan Nuclear Receptors and NF-κB Are Required for Appropriate Inflammatory Responses and Immune Cell Homeostasis. Biomolecules.

[66] Yamamoto T., Endo Y., Onodera A., Hirahara K., Asou H.K., Nakajima T., Kanno T., Ouchi Y., Uematsu S., Nishimasu H. (2018). DUSP10 constrains innate IL-33-mediated cytokine production in ST2hi memory-type pathogenic Th2 cells. Nat. Commun..

[67] Chung S., Ranjan R., Lee Y.G., Park G.Y., Karpurapu M., Deng J., Xiao L., Kim J.Y., Unterman T.G., Christman J.W. (2015). Distinct role of FoxO1 in M-CSF- and GM-CSF-differentiated macrophages contributes LPS-mediated IL-10: Implication in hyperglycemia. J. Leukoc. Biol..

[68] Elbjeirami W.M., Donnachie E.M., Burns A.R., Smith C.W. (2011). Endothelium-derived GM-CSF influences expression of oncostatin M. Am. J. Physiol. Cell Physiol..

[69] Haringman J.J., Gerlag D.M., Smeets T.J.M., Baeten D., van den Bosch F., Bresnihan B., Breedveld F.C., Dinant H.J., Legay F., Gram H. (2006). A randomized controlled trial with an anti-CCL2 (anti-monocyte chemotactic protein 1) monoclonal antibody in patients with RA. Arthritis Rheum..

[70] Tsai C.-H., Liu S.-C., Wang Y.-H., Su C.-M., Huang C.-C., Hsu C.-J., Tang C.-H. (2017). Osteopontin inhibition of miR-129-3p enhances IL-17 expression and monocyte migration in rheumatoid arthritis. Biochim. Biophys. Acta Gen. Subj..

[71] Wu Y.-J., Zhang S.-S., Yin Q., Lei M., Wang Q.-H., Chen W.-G., Luo T.-T., Zhou P., Ji C.-L. (2023). α-Mangostin Inhibited M1 Polarization of Macrophages/Monocytes in Antigen-Induced Arthritis Mice by Up-Regulating Silent Information Regulator 1 and Peroxisome Proliferators-Activated Receptor γ Simultaneously. Drug Des. Devel Ther..

[72] Vasilevko V., Ghochikyan A., Holterman M.J., Agadjanyan M.G. (2002). CD80 (B7-1) and CD86 (B7-2) are functionally equivalent in the initiation and maintenance of CD4+ T-cell proliferation after activation with suboptimal doses of PHA. DNA Cell Biol..

[73] Moghaddami M., Cleland L.G., Radisic G., Mayrhofer G. (2007). Recruitment of dendritic cells and macrophages during T cell-mediated synovial inflammation. Arthritis Res. Ther..

[74] König M., Rharbaoui F., Aigner S., Dälken B., Schüttrumpf J. (2016). Tregalizumab—A Monoclonal Antibody to Target Regulatory T Cells. Front. Immunol..

[75] Bossi G. (2015). Mutant p53 and sIL-1Ra. Aging.

[76] Wang M., Zhao H., Zhao H., Huo C., Yuan Y., Zhu Y. (2023). Moxibustion-mediated alleviation of synovitis in rats with rheumatoid arthritis through the regulation of NLRP3 inflammasome by modulating neutrophil extracellular traps. Heliyon.

[77] Jiang F.-Y., Zhang Y.-Z., Tai Y.-H., Chou C.-Y., Hsieh Y.-C., Chang Y.-C., Huang H.-C., Li Z.-Q., Hsieh Y.-C., Chen I.-J. (2023). A lesion-selective albumin-CTLA4Ig as a safe and effective treatment for collagen-induced arthritis. Inflamm. Regen..

[78] Tian Y., Ming J. (2022). Melatonin inhibits osteoclastogenesis via RANKL/OPG suppression mediated by Rev-Erbα in osteoblasts. J. Cell Mol. Med..

[79] Isozaki T., Nishimi S., Nishimi A., Saito M., Miwa Y., Toyoshima Y., Inagaki K., Kasama T. (2017). A disintegrin and metalloproteinase (ADAM)-10 as a predictive factor for tocilizumab effectiveness in rheumatoid arthritis. Mod. Rheumatol..

[80] Li S., Wang Y., Wu M., Younis M.H., Olson A.P., Barnhart T.E., Engle J.W., Zhu X., Cai W. (2022). Spleen-Targeted Glabridin-Loaded Nanoparticles Regulate Polarization of Monocyte/Macrophage (Mo/Mφ) for the Treatment of Cerebral Ischemia-Reperfusion Injury. Adv. Mater..

[81] Wang D.-D., He C.-Y., Wu Y.-J., Xu L., Shi C., Olatunji O.J., Zuo J., Ji C.-L. (2022). AMPK/SIRT1 Deficiency Drives Adjuvant-Induced Arthritis in Rats by Promoting Glycolysis-Mediated Monocytes Inflammatory Polarization. J. Inflamm. Res..

[82] Pang X., Yin P., Han J., Wang Z., Zheng F., Chen X. (2019). cPLA2a correlates with metastasis and poor prognosis of osteosarcoma by facilitating epithelial-mesenchymal transition. Pathol. Res. Pract..

[83] Lee A.Y.S., Eri R., Lyons A.B., Grimm M.C., Korner H. (2013). CC chemokine ligand 20 and its cognate receptor CCR6 in mucosal T cell immunology and inflammatory bowel disease: Odd couple or axis of evil?. Front. Immunol..

[84] Riella L.V., Dada S., Chabtini L., Smith B., Huang L., Dakle P., Mfarrej B., D‘Addio F., Adams L.-T., Kochupurakkal N. (2013). B7h (ICOS-L) maintains tolerance at the fetomaternal interface. Am. J. Pathol..

[85] Blagov A.V., Grechko A.V., Nikiforov N.G., Zhuravlev A.D., Sadykhov N.K., Orekhov A.N. (2022). Effects of Metabolic Disorders in Immune Cells and Synoviocytes on the Development of Rheumatoid Arthritis. Metabolites.

[86] Amoruso A., Sola D., Rossi L., Obeng J.A., Fresu L.G., Sainaghi P.P., Pirisi M., Brunelleschi S. (2016). Relation among anti-rheumatic drug therapy, CD14(+)CD16(+) blood monocytes and disease activity markers (DAS28 and US7 scores) in rheumatoid arthritis: A pilot study. Pharmacol. Res..

[87] Batko B., Schramm-Luc A., Skiba D.S., Mikolajczyk T.P., Siedlinski M. (2019). TNF-α Inhibitors Decrease Classical CD14hiCD16- Monocyte Subsets in Highly Active, Conventional Treatment Refractory Rheumatoid Arthritis and Ankylosing Spondylitis. Int. J. Mol. Sci..

[88] Du Z., Cong W., Tang K., Zheng Q., Song Z., Chen Y., Yang S., Zhang C., Ye T. (2023). Electroacupuncture stimulating Zusanli (ST36), Sanyinjiao (SP6) in mice with collagen-induced arthritis leads to adenosine A2A receptor-mediated alteration of p38α mitogen-activated protein kinase signaling and inhibition of osteoclastogenesis. J. Tradit. Chin. Med..

